# Supplementary material for: LSR-YOLO: A lightweight and fast model for retail products detection
Source: PLoS One. 2025 Oct 22;20(10):e0334216. doi: 10.1371/journal.pone.0334216 (PMC12543153; doi:10.1371/journal.pone.0334216)
Supplement: Attachment — Submitted filename: Final-PONE-D-25-15496-letter Response to Reviewers.docx [file pone.0334216.s002.docx]

Revision:

Manuscript ID PONE-D-25-15496 entitled "LSR-YOLO: a lightweight and fast YOLO model for the retail products detection"

| No | Authors’ response | |
| --- | --- | --- |
|  | We sincerely thank the editor and reviewers for their thorough evaluation of our manuscript and their valuable comments. We carefully considered and incorporated each of their suggestions, and made comprehensive revisions and improvements to the manuscript's format, content, figures, and formulas. These changes significantly improved the overall quality and readability of the work. Our point-by-point responses are provided as follows: | |
|  | Comments by editor | Authors’ response |
| 1 | A rebuttal letter that responds to each point raised by the academic editor and reviewer(s). You should upload this letter as a separate file labeled 'Response to Reviewers'. | We have prepared a rebuttal letter that responds to each point raised by the academic editor and reviewer(s), and named it A rebuttal letter Response to Reviewers. |
| 2 | A marked-up copy of your manuscript that highlights changes made to the original version. You should upload this as a separate file labeled 'Revised Manuscript with Track Changes'. | We have prepared a marked-up copy of your manuscript that highlights changes made to the original version and named it Revised Manuscript with Track Changes. |
| 3 | An unmarked version of your revised paper without tracked changes. You should upload this as a separate file labeled 'Manuscript'. | We have prepared an unmarked version of your revised paper without tracked changes. and named it 'Revised Manuscript'. |
| 4 | Please ensure that your manuscript meets PLOS ONE's style requirements, including those for file naming. | We used the template provided by your journal and made format modifications in accordance with the journal's requirements. Please see the Manuscript for details. |
| 5 | Please note that PLOS ONE has specific guidelines on code sharing for submissions in which author-generated code underpins the findings in the manuscript. In these cases, we expect all author-generated code to be made available without restrictions upon publication of the work. | We will coordinate to share the code we generate without any restrictions when publishing the work. We are also studying how to follow the guidelines you provided. Thank you. |
| 6 | Please update your submission to use the PLOS LaTeX template. | Our submitted manuscript has been prepared using Ms Word. If PLOS LaTeX template is must, we will transfer the manuscript into Latex in the next round. |
|  | Comments by Reviewer-1 | Authors’ response |
| 1 | The article proposes LSR-YOLO, a lightweight and fast object detection model based on YOLOv8n, optimized for retail product detection using techniques like DWConv, CSPHet-CBAM, ADown, and channel pruning, achieving a high inference speed of 357.1 FPS with a mAP50 of 72.2% on the Locount dataset. It demonstrates superior efficiency and accuracy compared to other models like YOLOv11, with reduced parameters (2,114,768) and GFLOPs (6.6), validated further on the COCO dataset for generalization.  Here are some major comments: - Figure 2 contains a typographical error where "Contact" is used instead of "Concat" to describe the concatenation operation in the improved YOLOv8n model architecture. Correcting this error and ensuring consistent terminology across all figures and text would enhance the article’s professionalism and clarity. | We are very sorry for the drawing error. We have corrected the text descriptions of each picture in the article.We have modified Figure 2. We respectfully inform you that certain details in Figure 2 have been refined, as highlighted by the red-boxed areas in the updated illustration. These refinements are intended solely to enhance the clarity of the figure, without affecting the results presented in the manuscript. We sincerely appreciate your understanding. The modified figure has also been modified and highlighted in line: **145 and 146** of the manuscript. In addition, we have improved the clarity of the figure.Please check the Figures.    **Figure 2.** Overall structure diagram of YOLOv8n-improved model for the retail product object detection. |
| 2 | The article could improve clarity by providing more detailed explanations of technical components like CSPHet-CBAM and ADown. For instance, a brief description of how CSPHet integrates with CBAM or how ADown's hybrid pooling strategy specifically enhances feature extraction would make the methodology more accessible to readers unfamiliar with these concepts. | We have provided a clearer explanation of the actual functions of each improvement point, with a focus on introducing the details of feature extraction and processing of each module, thereby enhancing the persuasiveness of the improved modules.  Specific modification is as follows:  (2.1.2. CSPHet-CBAM)--**Line: 191 to 215**  The CBAM module enhances feature extraction by merging two attention mechanisms: Channel Attention and Spatial Attention. These mechanisms enable the model to focus more effectively on key regions, particularly when identifying important items and local features, as shown in Figure 4. The Channel Attention adaptively assigns different weights to each channel, emphasizing the most critical features. In retail environments, where certain products may exhibit distinctive features in specific channels, the Channel Attention helps the model prioritize these significant details.  The Spatial Attention, on the other hand, generates a spatial attention map that reflects the spatial correlations among the features [35]. This allows the model to focus on critical regions of an object, especially in challenging scenarios where the object is partially occluded or surrounded by complex backgrounds. By combining these two attention mechanisms, CBAM improves the model's ability to selectively highlight and weight input features, enhancing its sensitivity to fine product details and making it more effective in detecting key items in cluttered environments.  In addition, Figure 5 introduces HetConv [36], an innovative convolutional structure that combines 1x1 and 3x3 convolution operations to effectively handle varying feature scales. Specifically, the 1x1 convolutions serve to reduce the number of channels, thereby decreasing the dimensionality and computational load. Meanwhile, the 3x3 convolutions capture local features and preserve spatial information, enabling the model to maintain a rich feature representation. By incorporating these two types of convolutions, HetConv strikes an effective balance between preserving important feature information and reducing computational complexity, which is particularly beneficial when processing high-dimensional data. This hybrid convolution approach not only retains essential details but also improves computational efficiency, making it well-suited for real-time applications such as retail product detection.  (2.1.3. ADown in backbone)--**Line :258 to 271**  In addition to its lightweight design, ADown employs a hybrid pooling strategy that integrates average pooling and max pooling. This combination is strategically used to capture a wider range of features from the input data. Average pooling, which typically focuses on smoothing and capturing background information, helps retain broader contextual features. On the other hand, max pooling focuses on identifying the most significant elements, excelling at preserving fine-grained texture and local features. By using both pooling techniques in tandem, ADown effectively enhances the model's ability to extract a more comprehensive and varied set of features, which improves the representation of the product data.  Moreover, ADown diverges from traditional convolutional architectures by using a parallel downsampling operation rather than a serial structure. This parallel approach ensures that more critical information is retained across layers while reducing spatial dimensions. This design choice improves computational efficiency and boosts the model's performance, particularly in real-time tasks like retail product detection. |
| 3 | The Locount dataset is central to the experiments, but its description is brief. Providing more details about its composition (e.g., types of retail products, image complexity, or annotation specifics) and why it was chosen over other retail datasets would strengthen the justification for its use and enhance reproducibility. | Based on your suggestions regarding the dataset, we have supplemented and explained the product types, annotations and other information of the Locount dataset. And we analyze in detail the reasons for choosing Locount over other datasets as the dataset for this study.  Specific modification is as follows: --Line: 357 to 371  (In section 3.1 )  The Locount dataset comprises a diverse collection of real-world images captured in retail environments, featuring a wide range of objects such as products, shelves, and customers. After refinement, it includes 109 categories of retail products, as depicted in Figure 8. The dataset is designed to support model training for detecting and counting items in crowded and cluttered retail settings.  And annotations are provided in a TXT format, which facilitates training across various object detection models. This dataset is particularly valuable for tasks such as inventory management, product recognition, and customer behavior analysis. The selection of the Locount dataset is driven by the specific needs of retail applications, ensuring it provides a realistic and challenging environment for model evaluation. The dataset has been carefully divided into a training set, validation set, and test set, containing 3,500, 1,000, and 500 images, respectively. The detailed composition of the dataset, including the types of retail products and the complexity of the images, as well as the reasoning behind its selection over other retail datasets, highlights its suitability for addressing real-world challenges in retail environments. |
| 4 | The article mentions trade-offs in accuracy due to model compression **but** does not discuss potential limitations or failure cases of LSR-YOLO, such as performance in extreme lighting conditions or with highly occluded objects. Including a section on limitations and challenges would provide a more balanced perspective. | We supplement the negative sample detection effect of the LSR-YOLO model under multi-target occlusion and weak lighting conditions, conducted a corresponding analysis, and presented the targeted research plan for the next step.  Specific modification is as follows:--**Line: 653 to 676**  (Section 3.8)  Balancing the trade-off between coverage of common scenarios and lightweight design, the LSR-YOLO model excels in standard visual environments and performs reasonably well in scenarios with motion blur and dense object arrangements. However, its detection performance is less effective in cases of object stacking and occlusion, as well as under low-light conditions.  As shown in the Figure 15, in the case of multiple bowls stacked and occluded, partial missed detections occur, and the bowls in the rear rows are not detected.    Figure 15. Examples of negative samples for product detection with multiple object occlusions.  In low-light conditions, as shown in Figure 16, the missed detection issue becomes more pronounced with LSR-YOLO. The model may have a reduced ability to perceive the color features of products with insufficient lighting, or the confidence scores may be significantly affected.    Figure 16. Examples of negative samples for product detection under low-light conditions.  In the next phase of the research, a targeted feature sensitivity analysis will be conducted on such occluded object samples, followed by further improvements to the model architecture. What’s more, corresponding training samples will be added to the dataset. For products under low-light conditions, light enhancement algorithms may be considered, or multimodal processing involving infrared cameras and visible light could be incorporated. |
| 5 | While Table 4 compares LSR-YOLO with other models, the discussion could be expanded to explain why modern models (e.g., Yolov8n, Yolov11n) perform poorly or how LSR-YOLO’s improvements specifically address their shortcomings. This would provide deeper insights into the proposed model’s advantages. | Response: We explain the detailed reason why modern models (e.g., Yolov8n, Yolov11n) perform poorly while LSR-YOLO perform better.  Specific modification as following:  (In section 3.5)--Line:528 to 543  Modern models such as YOLOv8n and YOLOv11 may struggle to strike a balance between computational complexity and inference speed, which becomes a bottleneck in practical retail applications. For example, while YOLOv8n achieves lower computational complexity, it suffers from relatively low accuracy. On the other hand, YOLOv8x offers higher accuracy but comes with significantly higher computational demands, resulting in slower inference speeds.  But LSR-YOLO specifically targets these shortcomings by implementing lightweight architectural improvements. The use of DWConv in the Neck, which separates convolution operations on the spatial and channel dimensions, significantly reduces parameter count and computational cost without sacrificing accuracy. Additionally, the introduction of the CSPHet-CBAM attention mechanism ensures that model accuracy remains high even after these architectural simplifications. Furthermore, by employing a channel pruning algorithm, LSR-YOLO eliminates less informative channels, further reducing computation and enhancing real-time performance, which is critical in retail environments where speed is essential. |
| 6 | The article should provide a detailed explanation for the significantly lower performance of YOLOv8n and YOLOv11 on the COCO2017 dataset compared to their original reported results, potentially addressing factors such as differences in training configurations, dataset preprocessing, or evaluation metrics to ensure transparency and contextualize the findings. | We have included additional details regarding the experimental prerequisites, such as hyperparameter settings and image input dimensions, to enhance the transparency of experiments on the COCO 2017 dataset. Specific modification is as follows: **Line:382. Line:566 to 569** (Section 3.6) The training setup is shown in Table 1. Using SGD optimizer, The model is trained with a batch size of 16, a learning rate of 0.01, and for 200 epochs. The input images are resized to 640×640 pixels by default, and momentum is set to 0.937 to accelerate convergence. **Table 1.** Experimental configuration. **Category** \| **Parameters** \| **Result**; Training parameters \| Operating System \| Ubuntu; \| Optimizer \| Stochastic gradient descent(SGD); \| Batch size \| 16; \| Learning rate \| 0.01; \| Epochs \| 200; \| Input images size \| 640 × 640; \| Momentum \| 0.937; Experimental environment parameters \| GPU \| NVIDIA GeForce RTX 3090; \| CPU \| Intel(R) Xeon(R) Platinum 8350C; \| CUDA \| 11.7; \| cuDNN \| 8.6; \| Pytorch \| 2.0.0; \| Python \| 3.8.17 |
|  | The article emphasizes deployment on edge devices but lacks details on real-world implementation challenges, such as hardware constraints or integration with retail robotic systems. Adding a brief discussion on practical deployment considerations would enhance the article’s applicability. | Considering the future deployment of the LSR-YOLO model in various robotics and edge computing devices, we conduct deployment trials on the low-power RK3568 lightweight device. The RK3568 chip features a quad-core 64-bit Cortex-A55 architecture with a clock speed of 2.0 GHz and integrates a Rockchip self-developed NPU, offering 1 TOPS of computational power. The LSR-YOLO model was first converted from the .pt format to .onnx, followed by inference testing.  Specific modification as following: **Line:677 to 687**  3.9. Preliminary deployment on Edge Computing Device  Considering the future application of the LSR-YOLO model in various robotics and edge computing devices, this study selected the low-power and inexpensive RK3568 device for deployment testing. The RK3568 chip features a quad-core 64-bit Cortex-A55 architecture with a clock speed of 2.0 GHz, and it integrates a Rockchip self-developed NPU, which reaches 1 TOPS. The LSR-YOLO model was first converted from the .pt format to .onnx, followed by inference testing. The final experimental results indicated that the model has 2,113,616 parameters, 3.80 GLOPs, and an inference speed of 10.6 FPS. As a low-performance edge device, these specifications meet the practical requirements for robotic inspection or handheld imaging devices, with higher-performance hardware expected to yield even better results. |
|  | Comments by Reviewer-2 | Authors’ response |
| 1 | I must say that the revisions made to the article have been executed exceptionally well. The changes have addressed all the concerns and suggestions that were previously raised, resulting in a much more polished and coherent piece of work. As I have thoroughly reviewed the revised version, I am satisfied with the improvements and clarity it now possesses. Therefore, I have no further questions or additional points of clarification regarding this article. | Thank you for recognizing the value of this study. I will incorporate the feedback from other reviewers to revise the manuscript and enhance its overall quality. |
|  | Comments by Reviewer-3 | Authors’ response |
| 1 | This manuscript presents LSR-YOLO, an improved and lightweight version of YOLOv8n tailored for real-time retail product detection. The model integrates architectural modifications including DWConv, CSPHet-CBAM attention, and ADown pooling for efficiency. The authors further apply structured channel pruning to compress the model, achieving significant gains in speed and parameter reduction without compromising detection accuracy. Extensive evaluation on the Locount and COCO datasets supports the claims. follwoing comments may be addressed before publication at plosOne.  The individual novelty of components (DWConv, CBAM, ADown) is limited as each is previously known; the contribution lies in their integration and optimization for retail. | Thank you for your thoughtful feedback. While it is true that components such as DWConv, CBAM, and ADown have been explored individually in prior work, the novelty of our approach lies not only in their integration but also in how these components are optimized and applied specifically for retail product detection. Each of these components has been carefully selected and fine-tuned to address the unique challenges of the retail environment, such as handling varying feature scales and complex backgrounds.  At the same time, we have added many detailed explanations of improvements, which can better reflect the practical effect of the method proposed in this paper and consider the retail scenarios.  Specific modification as following:  (2.1.2. CSPHet-CBAM)--**Line:191 to 215**  The CBAM module enhances feature extraction by merging two attention mechanisms: Channel Attention and Spatial Attention. These mechanisms enable the model to focus more effectively on key regions, particularly when identifying important items and local features, as shown in Figure 4. The Channel Attention adaptively assigns different weights to each channel, emphasizing the most critical features. In retail environments, where certain products may exhibit distinctive features in specific channels, the Channel Attention helps the model prioritize these significant details.  The Spatial Attention, on the other hand, generates a spatial attention map that reflects the spatial correlations among the features [35]. This allows the model to focus on critical regions of an object, especially in challenging scenarios where the object is partially occluded or surrounded by complex backgrounds. By combining these two attention mechanisms, CBAM improves the model's ability to selectively highlight and weight input features, enhancing its sensitivity to fine product details and making it more effective in detecting key items in cluttered environments.  In addition, Figure 5 introduces HetConv [36], an innovative convolutional structure that combines 1x1 and 3x3 convolution operations to effectively handle varying feature scales. Specifically, the 1x1 convolutions serve to reduce the number of channels, thereby decreasing the dimensionality and computational load. Meanwhile, the 3x3 convolutions capture local features and preserve spatial information, enabling the model to maintain a rich feature representation. By incorporating these two types of convolutions, HetConv strikes an effective balance between preserving important feature information and reducing computational complexity, which is particularly beneficial when processing high-dimensional data. This hybrid convolution approach not only retains essential details but also improves computational efficiency, making it well-suited for real-time applications such as retail product detection.  **Line:221 to 241**  And it provides a highly flexible and efficient solution to capture multi-scale features. This hybrid convolution approach is distinct from previous works that use fixed or uniform convolution operations, as it dynamically adapts to varying feature scales, reducing computational complexity without sacrificing accuracy, particularly when handling high-dimensional data in settings with many channels.  Furthermore, the integration of CBAM within the CSPHet-CBAM framework introduces a dual attention mechanism (channel and spatial attention) that is designed not just for general feature enhancement, but specifically for distinguishing critical object features in cluttered environments. Unlike previous methods for retail products that apply attention mechanisms independently, CSPHet-CBAM strategically combines both attention types to improve the model's focus on essential details, especially under challenging conditions like partial occlusions or backgrounds with high noise. The channel attention mechanism allows the model to dynamically emphasize the most relevant feature channels, while the spatial attention mechanism enables the model to concentrate on significant spatial regions, boosting its performance in object detection tasks with intricate backgrounds.  The Cross-Stage Partial (CSP) design further distinguishes CSPHet-CBAM by incorporating partial residual connections that facilitate more efficient feature flow across stages. This design mitigates the gradient vanishing problem by ensuring smoother gradient propagation, improving model convergence, and increasing robustness, especially in deep architectures.  (2.1.3. ADown in backbone)--**Line:258 to 271**  In addition to its lightweight design, ADown employs a hybrid pooling strategy that integrates average pooling and max pooling. This combination is strategically used to capture a wider range of features from the input data. Average pooling, which typically focuses on smoothing and capturing background information, helps retain broader contextual features. On the other hand, max pooling focuses on identifying the most significant elements, excelling at preserving fine-grained texture and local features. By using both pooling techniques in tandem, ADown effectively enhances the model's ability to extract a more comprehensive and varied set of features, which improves the representation of the product data.  Moreover, ADown diverges from traditional convolutional architectures by using a parallel downsampling operation rather than a serial structure. This parallel approach ensures that more critical information is retained across layers while reducing spatial dimensions. This design choice improves computational efficiency and boosts the model's performance, particularly in real-time tasks like retail product detection. |
| 2 | Comparison with other lightweight models (e.g., MobileNetV3, EfficientDet-Lite, YOLO-NAS) is missing and could contextualize performance better. | We supplemented the lightweight network MobileNetV3 and YOLO-NAS as the comparative experimental models. Specific modification is as follows**:--Line:507 to 513** **Table 5.** Comparison of Different Object Detection Algorithms on Locount dataset. **Model** \| **Result** \| \| \| \| \| \|; \| **Precision** \| **Recall** \| **mAP50** \| **mAP50-95** \| **Params** \| **GFLOPs** \| **FPS**; SSD 300 [16] \| 0.623 \| 0.435 \| 0.548 \| 0.366 \| 4818100 \| 35.8 \| 121.6; Faster-RCNN [24] \| 0.613 \| 0.422 \| 0.538 \| 0.351 \| 41902000 \| 267.3 \| 87.7; YOLOv5 [49-50] \| 0.585 \| 0.51 \| 0.579 \| 0.412 \| **1906642** \| 14.6 \| 95.2; YOLOv7 [47] \| 0.738 \| 0.584 \| 0.581 \| 0.429 \| 37064324 \| 10.5 \| 119; YOLOv8n [26] \| 0.708 \| 0.502 \| 0.600 \| 0.395 \| 3343451 \| 13.1 \| 110.4; Swin Transformer [51] \| 0.772 \| 0.376 \| 0.571 \| 0.337 \| 45300000 \| 213.0 \| 72.9; RT-DETR [52] \| 0.753 \| 0.361 \| 0.563 \| 0.447 \| 32207735 \| 103.9 \| 28.4; MobileNetV3 [53] \| 0.462 \| 0.498 \| 0.588 \| 0.423 \| 10617000 \| 166.0 \| 57.8; YOLO-NAS [54] \| 0.146 \| 0.792 \| 0.572 \| 0.398 \| 19066967 \| 17.2 \| 29.4; YOLOv11 [48] \| 0.552 \| 0.538 \| 0.58 \| 0.411 \| 2650315 \| 6.7 \| 340.3; **YOLOv8n-improved** \| 0.71 \| 0.502 \| 0.592 \| 0.379 \| 2574947 \| 8.1 \| 277.7; **LSR-YOLO** \| **0.741** \| **0.649** \| **0.722** \| **0.478** \| 2114768 \| **6.6** \| **357.1** It is worth noting that transformer-based models face the issue of excessive computational complexity, which is likely attributed to their too deep network layers. Meanwhile, existing lightweight models such as YOLO-NAS, despite having fewer parameters, suffer from slow inference speeds, rendering them impractical for real-time retail applications. **--Line:614** **Table 7.** Comparison of detection performance between LSR-YOLO and other models on COCO2017 dataset. **Model** \| **Result** \| \| \| \| \| \|; \| **Precision** \| **Recall** \| **mAP50** \| **mAP50-95** \| **Params** \| **GFLOPs** \| **FPS**; SSD [16] \| 0.632 \| 0.341 \| 0.398 \| 0.217 \| 34305000 \| 34.3 \| 110.1; Faster-RCNN [24] \| 0.660 \| 0.383 \| 0.486 \| 0.325 \| 41753000 \| 187 \| 31.8; YOLOv5 [49-50] \| 0.583 \| 0.442 \| 0.487 \| 0.329 \| 7225885 \| 16.4 \| 289.4; YOLOv7 [47] \| 0.570 \| 0.482 \| 0.483 \| 0.324 \| 6221370 \| 13.7 \| 185.1; YOLOv8n[26] \| 0.604 \| 0.446 \| 0.493 \| **0.342** \| 3151904 \| 9.6 \| 112.3; Swin Transformer [51] \| 0.365 \| 0.420 \| 0.447 \| 0.254 \| 45300000 \| 194.0 \| 10.2; RT-DETR [52] \| 0.794 \| 0.144 \| 0.470 \| 0.415 \| 32148140 \| 103.8 \| 91.7; MobileNetV3 [53] \| 0.398 \| 0.406 \| 0.441 \| 0.289 \| 10016000 \| 136.0 \| 26.7; YOLO-NAS [54] \| 0.034 \| 0.756 \| 0.446 \| 0.305 \| 19053888 \| 17.2 \| 45.8; YOLOv11 [48] \| 0.601 \| 0.442 \| 0.484 \| **0.346** \| 2616248 \| 6.5 \| 318.7; **YOLOv8n-improved** \| 0.577 \| 0.438 \| 0.490 \| 0.335 \| 2383400 \| 7.2 \| 322.5; **LSR-YOLO** \| 0.564 \| 0.439 \| **0.495** \| 0.341 \| **1768097** \| **5.1** \| **333.3** |
| 3 | Authors didnot performed confidence intervals or statistical significance testing across multiple runs. | The object confidence threshold for detection is 0.001 when training, which is the default value from Ultralytics team.  Specific modification as following:--**Line:410 to 414**  (Section 3.3)  During model training, the confidence threshold for the target object is set to the default value of 0.001, ensuring that small, difficult-to-detect targets are adequately considered as far as possible. The training is conducted using the default of 100 epochs from Ultralytics, ensuring the capture of the model's optimal performance, with results that can be consistently reproduced. |
| 4 | Please add training curves to give better look | We supplemented the training curve before model compression to ensure the completeness of the experimental result presentation.  Specific modification is as follows:--**Line:410 to 416**  See Figure 9  During model training, the confidence threshold for the target object is set to the default value of 0.001, ensuring that small, difficult-to-detect targets are adequately considered as far as possible. The hyperparameters are shown in Table 1. The training is conducted using the default of 100 epochs from Ultralytics, ensuring the capture of the model's optimal performance, with results that can be consistently reproduced.    **Figure 9.** The training curves of metrics in YOLOv8n-improved model. |
| 5 | There are occasional grammatical errors. Do a thorugh proof read before revision submission. For example:“...processing at 357.1 fps compared to YOLOv8n baseline’s 78.8 fps...” → should be 78.8 fps for consistency.  you should use consistent notation: “YOLOv8n-improved” vs. “improved YOLOv8n”. | We corrected the grammatical errors throughout the article and refined and revised the writing in light of other suggestions.  On the other hand, we are particularly sorry，Due to improperly set training parameters, the YOLOv8n baseline's 78.8 fps was incorrectly obtained. This was incorrectly obtained, and after multiple training runs, the correct YOLOv8n baseline's 110.4 fps was obtained. We have now corrected this error and apologize for the error. Thank you.  Specific modification is as follows:  YOLOv8n-improved  etc. |
|  | Comments by Reviewer-4 | Authors’ response |
| 1 | The manuscript introduces LSR-YOLO, a lightweight and efficient object detection model tailored for real-time retail applications. The model builds upon YOLOv8n and incorporates three key enhancements—DWConv, CSPHet-CBAM attention module, and ADown for downsampling—followed by structured channel pruning. The proposed approach is compelling and well-validated through multiple experiments.  Strengths: The manuscript addresses a relevant and practical challenge in real-time retail object detection. Detailed methodology with clear architectural diagrams (DWConv, CBAM, ADown). Strong empirical evaluation using appropriate metrics and datasets (Locount and COCO). Clear improvement over baseline models in terms of both speed and accuracy. Thoughtful ablation and pruning analysis enhance the credibility of the findings.  Suggestions for Improvement: Language: Numerous grammatical errors and stylistic inconsistencies must be corrected. Consider professional editing. | Thank you for your valuable feedback. We have thoroughly reviewed the manuscript and addressed the grammatical errors and stylistic inconsistencies. The text has been revised to more clear and more professional. |
| 2 | Clarity: The description of some modules (e.g., CSPHet-CBAM) is dense; more intuitive explanations or simplified illustrations could help. | We made targeted modifications to the descriptions of each improvement point, directly highlighting the role played by each detail and eliminating redundant statements.  Specific modification is as follows:  (Section 2.1.2) --**Line:221to 236**  And it provides a highly flexible and efficient solution to capture multi-scale features. This hybrid convolution approach is distinct from previous works that use fixed or uniform convolution operations, as it dynamically adapts to varying feature scales, reducing computational complexity without sacrificing accuracy, particularly when handling high-dimensional data in settings with many channels.  Furthermore, the integration of CBAM within the CSPHet-CBAM framework introduces a dual attention mechanism (channel and spatial attention) that is designed not just for general feature enhancement, but specifically for distinguishing critical object features in cluttered environments. Unlike previous methods for retail products that apply attention mechanisms independently, CSPHet-CBAM strategically combines both attention types to improve the model's focus on essential details, especially under challenging conditions like partial occlusions or backgrounds with high noise. The channel attention mechanism allows the model to dynamically emphasize the most relevant feature channels, while the spatial attention mechanism enables the model to concentrate on significant spatial regions, boosting its performance in object detection tasks with intricate backgrounds.  etc. |
| 3 | References: Many citations are to arXiv or conference proceedings without DOI or full publication details. Ensure formatting aligns with journal standards. | Response: We add DOI for arXiv or conference proceedings, giving the details.  Specific modification as following:  [5] Hashemi N S, Aghdam R B, Ghiasi A S B, et al. Template matching advances and applications in image analysis. arXiv preprint arXiv:1610.07231, 2016. <https://doi.org/10.48550/arXiv.1610.07231>  [8] Kaur P, Kaur B. 2-D geometric shape recognition using canny edge detection technique. 2016 3rd International Conference on Computing for Sustainable Global Development (INDIACom). IEEE, 2016, 3161-3164. [ieee.org/document/7724847](https://ieeexplore.ieee.org/document/7724847)  [13] O'Shea K. An introduction to convolutional neural networks. arXiv preprint arXiv:1511.08458, 2015. <https://doi.org/10.48550/arXiv.1511.08458>  [16] Liu W, Anguelov D, Erhan D, et al. Ssd: Single shot multibox detector. Computer Vision–ECCV 2016: 14th European Conference, Amsterdam, The Netherlands, October 11–14, 2016, Proceedings, Part I 14. Springer International Publishing, 2016, 21-37. <https://doi.org/10.1007/978-3-319-46448-0_2>  [18] Bochkovskiy A, Wang C Y, Liao H Y M. Yolov4: Optimal speed and accuracy of object detection. arXiv preprint arXiv:2004.10934, 2020. <https://doi.org/10.48550/arXiv.2004.10934>  [19] Tan M, Pang R, Le Q V. Efficientdet: Scalable and efficient object detection. Proceedings of the IEEE/CVF conference on computer vision and pattern recognition. 2020, 10781-10790. <https://doi.org/10.1109/CVPR42600.2020.01079>  [20] Bai Y, Chen Y, Yu W, et al. Products-10k: A large-scale product recognition dataset. arXiv preprint arXiv:2008.10545, 2020. <https://doi.org/10.48550/arXiv.2008.10545>  [21] Cai Y, Wen L, Zhang L, et al. Rethinking object detection in retail stores. Proceedings of the AAAI Conference on Artificial Intelligence. 2021, 35(2), 947-954. <https://doi.org/10.1609/aaai.v35i2.16178>  [22] Lin T Y, Maire M, Belongie S, et al. Microsoft coco: Common objects in context. Computer Vision–ECCV 2014: 13th European Conference, Zurich, Switzerland, September 6-12, 2014, Proceedings, Part V 13. Springer International Publishing, 2014, 740-755. <https://doi.org/10.1007/978-3-319-10602-1_48>  [25] He K, Gkioxari G, Dollár P, et al. Mask r-cnn. Proceedings of the IEEE international conference on computer vision. 2017, 2961-2969. [10.1109/ICCV.2017.322](https://doi.org/10.1109/ICCV.2017.322)  [29] Guo Y, Li Y, Wang L, et al. Depthwise convolution is all you need for learning multiple visual domains. Proceedings of the AAAI Conference on Artificial Intelligence. 2019, 33(01), 8368-8375. [10.1609/aaai.v33i01.33018368](https://doi.org/10.1609/aaai.v33i01.33018368)  [31] Hendrycks D, Gimpel K. Gaussian error linear units (GELUs). arXiv preprint arXiv:1606.08415, 2016. <https://doi.org/10.48550/arXiv.1606.08415>  [34] Woo S, Park J, Lee J Y, et al. Cbam: Convolutional block attention module. Proceedings of the European conference on computer vision (ECCV). 2018, 3-19. <https://doi.org/10.1007/978-3-030-01234-2_1>  [35] Zhu X, Cheng D, Zhang Z, et al. An empirical study of spatial attention mechanisms in deep networks. Proceedings of the IEEE/CVF international conference on computer vision. 2019, 6688-6697. <https://doi.org/10.1109/ICCV.2019.00679>  [36] Singh P, Verma V K, Rai P, et al. Hetconv: Heterogeneous kernel-based convolutions for deep cnns. Proceedings of the IEEE/CVF conference on computer vision and pattern recognition. 2019, 4835-4844. <https://doi.org/10.1109/CVPR.2019.00497>  [37] Wang C Y, Yeh I H, Mark Liao H Y. Yolov9: Learning what you want to learn using programmable gradient information[C]. European conference on computer vision. Cham: Springer Nature Switzerland, 2024, 1-21. <https://link.springer.com/chapter/10.1007/978-3-031-72751-1_1>  [39] Christlein V, Spranger L, Seuret M, et al. Deep generalized max pooling. 2019 International conference on document analysis and recognition (ICDAR). IEEE, 2019, 1090-1096. <https://doi.org/10.1109/ICDAR.2019.00177>  [41] Gale T, Elsen E, Hooker S. The state of sparsity in deep neural networks. arXiv preprint arXiv:1902.09574, 2019. <https://doi.org/10.48550/arXiv.1902.09574>  [46] Desislavov R, Martínez-Plumed F, Hernández-Orallo J. Trends in AI inference energy consumption: Beyond the performance-vs-parameter laws of deep learning[J]. Sustainable Computing: Informatics and Systems (2023). Volume 38, April 2023, 100857.  [47] Wang C Y, Bochkovskiy A, Liao H Y M. YOLOv7: Trainable bag-of-freebies sets new state-of-the-art for real-time object detectors. Proceedings of the IEEE/CVF conference on computer vision and pattern recognition. 2023, 7464-7475. <https://doi.org/10.1109/CVPR52729.2023.00721>  [48] Khanam R, Hussain M. Yolov11: An overview of the key architectural enhancements. arXiv preprint arXiv:2410.17725, 2024. <https://doi.org/10.48550/arXiv.2410.17725>  [50] Li G, Xie Y, Lu Y, et al. Enhancing precision object detection and identification for autonomous vehicles through YOLOv5 refinement with YOLO-ALPHA[C]. Proc. Int. Conf. Artif. Life Rob. 2024, 2:889-894. [ICAROB.2024.OS26-11](http://dx.doi.org/10.5954/ICAROB.2024.OS26-11)  [51] Liu Z, Lin Y, Cao Y, et al. Swin transformer: Hierarchical vision transformer using shifted windows[C]. Proceedings of the IEEE/CVF international conference on computer vision. 2021: 10012-10022. [ICCV48922.2021.00986](https://doi.org/10.1109/ICCV48922.2021.00986)  [52] Zhao Y, Lv W, Xu S, et al. Detrs beat yolos on real-time object detection[C]. Proceedings of the IEEE/CVF conference on computer vision and pattern recognition. 2024: 16965-16974. <https://doi.org/10.1109/CVPR52733.2024.01605>  [53] Koonce B. MobileNetV3[M]. Convolutional neural networks with swift for tensorflow: image recognition and dataset categorization. Berkeley, CA: Apress, 2021: 125-144.  [54] Terven J, Córdova-Esparza D M, Romero-González J A. A comprehensive review of yolo architectures in computer vision: From yolov1 to yolov8 and yolo-nas[J]. Machine learning and knowledge extraction, 2023, 5(4): 1680-1716. |
| 4 | Figures: Figures mentioned (e.g., Figure 1 to Figure 12) should be made visible in the manuscript file to fully evaluate their clarity and contribution. | We adjust the resolution of each figure to make them appear clearer.  Specific modification is as follows:  Please check Figure 1 to Figure 13. |
| 5 | Model limitations: Discuss potential trade-offs or limitations, such as potential accuracy drops under severe occlusion or small object detection. | We add the negative sample detection effect of the LSR-YOLO model under multi-target occlusion and weak lighting conditions, conducted a corresponding analysis, and presented the targeted research plan to handle limitations.  Specific modification is as follows:  (Section 3.8)**--Line:654 to 676**  Balancing the trade-off between coverage of common scenarios and lightweight design, the LSR-YOLO model excels in standard visual environments and performs reasonably well in scenarios with motion blur and dense object arrangements. However, its detection performance is less effective in cases of object stacking and occlusion, as well as under low-light conditions.  As shown in the Figure 15, in the case of multiple bowls stacked and occluded, partial missed detections occur, and the bowls in the rear rows are not detected.    **Figure 15.** Examples of negative samples for product detection with multiple object occlusions.  In low-light conditions, as shown in Figure 16, the missed detection issue becomes more pronounced with LSR-YOLO.The model may have a reduced ability to perceive the color features of products with insufficient lighting, or the confidence scores may be significantly affected.    **Figure 16.** Examples of negative samples for product detection under low-light conditions.  In the next phase of the research, a targeted feature sensitivity analysis will be conducted on such occluded object samples, followed by further improvements to the model architecture. What’s more, corresponding training samples will be added to the dataset. For products under low-light conditions, light enhancement algorithms may be considered, or multimodal processing involving infrared cameras and visible light could be incorporated. |
|  | Comments by Reviewer-5 | Authors’ response |
| 1 | Limited novelty in methodology: While CSPHet-CBAM is presented as novel, it is primarily a hybrid of known techniques (CBAM + HetConv).  Recommend the authors clarify how this differs from past works that use similar hybrid attention or mixed convolution strategies. | Thank you for the insightful feedback. While it is true that CSPHet-CBAM incorporates well-established components such as CBAM and HetConv, its novelty lies in the unique manner in which these techniques are hybridized and optimized for retail-specific applications, leading to enhanced performance in real-world scenarios. We recognize that attention mechanisms and convolution strategies have been explored extensively in prior works; however, the integration and optimization of these techniques in CSPHet-CBAM are tailored to address key challenges in retail, such as small object detection and complex background handling.  For this, we have revised the previous description, highlighting the innovativeness of the improvement points  Specific modification is as follows:  (In Section 2.1.2. CSPHet-CBAM)--Line:221to 241  And it provides a highly flexible and efficient solution to capture multi-scale features. This hybrid convolution approach is distinct from previous works that use fixed or uniform convolution operations, as it dynamically adapts to varying feature scales, reducing computational complexity without sacrificing accuracy, particularly when handling high-dimensional data in settings with many channels.  Furthermore, the integration of CBAM within the CSPHet-CBAM framework introduces a dual attention mechanism (channel and spatial attention) that is designed not just for general feature enhancement, but specifically for distinguishing critical object features in cluttered environments. Unlike previous methods for retail products that apply attention mechanisms independently, CSPHet-CBAM strategically combines both attention types to improve the model's focus on essential details, especially under challenging conditions like partial occlusions or backgrounds with high noise. The channel attention mechanism allows the model to dynamically emphasize the most relevant feature channels, while the spatial attention mechanism enables the model to concentrate on significant spatial regions, boosting its performance in object detection tasks with intricate backgrounds.  The Cross-Stage Partial (CSP) design further distinguishes CSPHet-CBAM by incorporating partial residual connections that facilitate more efficient feature flow across stages. This design mitigates the gradient vanishing problem by ensuring smoother gradient propagation, improving model convergence, and increasing robustness, especially in deep architectures. |
| 2 | Overemphasis on FPS: The manuscript prioritizes FPS gains, but real-world inference latency, energy consumption, or deployment on actual hardware (e.g., Jetson Nano, Raspberry Pi) would strengthen the practical relevance. | We selected the low-power and inexpensive RK3568 device for deployment testing. The RK3568 chip features a quad-core 64-bit Cortex-A55 architecture with a clock speed of 2.0 GHz, and it integrates a Rockchip self-developed NPU, which reaches 1 TOPS.  Specific modification is as follows: **--Line:677 to 687**  3.9. Preliminary deployment on Edge Computing Device  Considering the future application of the LSR-YOLO model in various robotics and edge computing devices, this study selected the low-power and inexpensive RK3568 device for deployment testing. The RK3568 chip features a quad-core 64-bit Cortex-A55 architecture with a clock speed of 2.0 GHz, and it integrates a Rockchip self-developed NPU, which reaches 1 TOPS. The LSR-YOLO model was first converted from the .pt format to .onnx, followed by inference testing. The final experimental results indicated that the model has 2,113,616 parameters, 3.80 GLOPs, and an inference speed of 10.6 FPS. As a low-performance edge device, these specifications meet the practical requirements for robotic inspection or handheld imaging devices, with higher-performance hardware expected to yield even better results. |
| 3 | Pruning Analysis Needs Depth: Sparse training is covered, but more discussion on pruning criteria (e.g., L1 norm threshold choice) and stability across runs would improve robustness claims. | We have provided detailed supplementary explanations on the specifics of sparse training. Moreover, the stability of sparse training is extremely high. After setting the pruning strategy, there will be no jumps in multiple results.  Specific modification is as follows:**--Line:337 to 342**  (In section 2.2)  The formula for the sparsification training loss function is as follows:  $\text{L=}\sum_{\text{(x,y)}} \text{(f}\left( \text{x, W} \right)\text{,y)}\text{L}^{\text{'}}\text{+λ}\sum_{\text{γϵΓ}} \text{g(γ)}$ (9)  where $\text{x}$, $\text{y}$, $\text{W}$ and $\text{f}$ represent the input, target, weights, and output function of the original network, respectively. $\text{λ}$ is the sparsity factor, $\text{g(γ)}$ denotes the penalty function applied to the scaling factor γ, and $\text{L}^{\text{'}}$ denotes the loss function of the original network. By multiple tests, $\text{λ}$ is set as an empirical value of 0.005, providing a reasonable trade-off between sparsity and the preservation of essential model information. |
| 4 | Missing Comparisons with Transformer-based Detectors: While YOLO-family comparisons are extensive, the paper does not consider newer lightweight transformer-based models (e.g., YOLOS, Mobile-DETR). A discussion of these would be valuable. | In response to your feedback, we have add two representative Transformer-based object detection models (Swin Transformer and RT-DETR) that are widely recognized for their stability and practical use, and we have provided a corresponding analysis. Specific modification is as follows: (Section 3.5)**--Line:528 to 534** **Table 5.--Line:507** **Model** \| **Result** \| \| \| \| \| \|; \| **Precision** \| **Recall** \| **mAP50** \| **mAP50-95** \| **Params** \| **GFLOPs** \| **FPS**; SSD 300 [16] \| 0.623 \| 0.435 \| 0.548 \| 0.366 \| 4818100 \| 35.8 \| 121.6; Faster-RCNN [24] \| 0.613 \| 0.422 \| 0.538 \| 0.351 \| 41902000 \| 267.3 \| 87.7; YOLOv5 [49-50] \| 0.585 \| 0.51 \| 0.579 \| 0.412 \| **1906642** \| 14.6 \| 95.2; YOLOv7 [47] \| 0.738 \| 0.584 \| 0.581 \| 0.429 \| 37064324 \| 10.5 \| 119; YOLOv8n [26] \| 0.708 \| 0.502 \| 0.600 \| 0.395 \| 3343451 \| 13.1 \| 110.4; Swin Transformer [51] \| 0.772 \| 0.376 \| 0.571 \| 0.337 \| 45300000 \| 213.0 \| 72.9; RT-DETR [52] \| 0.753 \| 0.361 \| 0.563 \| 0.447 \| 32207735 \| 103.9 \| 28.4; MobileNetV3 [53] \| 0.462 \| 0.498 \| 0.588 \| 0.423 \| 10617000 \| 166.0 \| 57.8; YOLO-NAS [54] \| 0.146 \| 0.792 \| 0.572 \| 0.398 \| 19066967 \| 17.2 \| 29.4; YOLOv11 [48] \| 0.552 \| 0.538 \| 0.58 \| 0.411 \| 2650315 \| 6.7 \| 340.3; **YOLOv8n-improved** \| 0.71 \| 0.502 \| 0.592 \| 0.379 \| 2574947 \| 8.1 \| 277.7; **LSR-YOLO** \| **0.741** \| **0.649** \| **0.722** \| **0.478** \| 2114768 \| **6.6** \| **357.1** Modern models such as YOLOv8n and YOLOv11 may struggle to strike a balance between computational complexity and inference speed, which becomes a bottleneck in practical retail applications. For example, while YOLOv8n achieves lower computational complexity, it suffers from relatively low accuracy. On the other hand, YOLOv8x offers higher accuracy but comes with significantly higher computational demands, resulting in slower inference speeds. **(Section 3.6.2)--Line:611 to 613** Meanwhile, Transformer-based models continue to face challenges related to excessive computational demands and low FPS. **Table 7.--Line:614** **Model** \| **Result** \| \| \| \| \| \|; \| **Precision** \| **Recall** \| **mAP50** \| **mAP50-95** \| **Params** \| **GFLOPs** \| **FPS**; SSD [16] \| 0.632 \| 0.341 \| 0.398 \| 0.217 \| 34305000 \| 34.3 \| 110.1; Faster-RCNN [24] \| 0.660 \| 0.383 \| 0.486 \| 0.325 \| 41753000 \| 187 \| 31.8; YOLOv5 [49-50] \| 0.583 \| 0.442 \| 0.487 \| 0.329 \| 7225885 \| 16.4 \| 289.4; YOLOv7 [47] \| 0.570 \| 0.482 \| 0.483 \| 0.324 \| 6221370 \| 13.7 \| 185.1; YOLOv8n[26] \| 0.604 \| 0.446 \| 0.493 \| **0.342** \| 3151904 \| 9.6 \| 112.3; Swin Transformer [51] \| 0.365 \| 0.420 \| 0.447 \| 0.254 \| 45300000 \| 194.0 \| 10.2; RT-DETR [52] \| 0.794 \| 0.144 \| 0.470 \| 0.415 \| 32148140 \| 103.8 \| 91.7; MobileNetV3 [53] \| 0.398 \| 0.406 \| 0.441 \| 0.289 \| 10016000 \| 136.0 \| 26.7; YOLO-NAS [54] \| 0.034 \| 0.756 \| 0.446 \| 0.305 \| 19053888 \| 17.2 \| 45.8; YOLOv11 [48] \| 0.601 \| 0.442 \| 0.484 \| **0.346** \| 2616248 \| 6.5 \| 318.7; **YOLOv8n-improved** \| 0.577 \| 0.438 \| 0.490 \| 0.335 \| 2383400 \| 7.2 \| 322.5; **LSR-YOLO** \| 0.564 \| 0.439 \| **0.495** \| 0.341 \| **1768097** \| **5.1** \| **333.3** |
| 5 | No Variance or Confidence Reporting: The results are reported as point estimates (e.g., “Precision = 0.741”), but no standard deviation, confidence interval, or multiple-run averages are reported.  This is a concern, especially since deep learning training can produce different results depending on initialization or data shuffling. | We add the introduction to the confidence level and emphasized the Settings of the experimental parameters in this paper. Later, we add cross-validation (section 3.7) to prove the reproducibility of the model. Specific modification is as follows: (Section 3.3)**--Line:410 to 414** During model training, the confidence threshold for the target object is set to the default value of 0.001, ensuring that small, difficult-to-detect targets are adequately considered as far as possible. The hyperparameters are shown in Table 1. (Section 3.7)**--Line:638 to 641** For the training and validation sets comprising a total of 4500 images, 5-fold cross-validation is performed to obtain more reliable results. The final results of the cross-validation experiments are presented in the Table 8. **Table 8.** The 5-Fold Cross Validation result of LSR-YOLO on Locount dataset.--**Line:643** **Model** \| **Precision** \| **Recall** \| **mAP50** \| **mAP50-95** \| **Parameters** \| **CFLOPs** \| **FPS**; LSR-YOLO \| 0.741 \| 0.649 \| 0.722 \| 0.478 \| 2114768 \| 6.6 \| 357.1; **5-Fold CV** \| 0.749 \| 0.609 \| 0.680 \| 0.493 \| 2485861 \| 7.8 \| 349.7 |
| 6 | No Statistical Tests: There is no use of statistical hypothesis testing (e.g., t-tests, ANOVA) to determine if improvements over baseline models are statistically significant.  While not always required in ML papers, it would strengthen the claim that LSR-YOLO consistently outperforms others. | Thank you for your thoughtful comment. While we understand the value of statistical hypothesis testing in certain contexts, we would like to clarify that such tests, including t-tests and ANOVA, are not typically well-suited for deep learning experiments. The highly complex and non-linear nature of deep learning models often leads to inherent variability in results due to factors such as training conditions, data distribution, and random initialization. And statistical hypothesis testing might not provide meaningful insights in this context. |
| 7 | No Cross-Validation: The model is evaluated using a fixed train-validation-test split. Cross-validation or even repeating experiments with different splits would give more reliable performance estimates. | We supplement with a 5-Fold cross validation. The results showed that the changes in each indicator were very small, indicating that the model has strong robustness and is not highly dependent on specific data. Specific modification is as follows: 3.7. 5-Fold Cross Validation----**Line:638 to 652** For the training and validation sets comprising a total of 4500 images, 5-fold cross-validation is performed to obtain more reliable results. The final results of the cross-validation experiments are presented in the Table 8. **Table 8.** The 5-Fold Cross Validation result of LSR-YOLO on Locount dataset.--**Line:643** **Model** \| **Precision** \| **Recall** \| **mAP50** \| **mAP50-95** \| **Parameters** \| **CFLOPs** \| **FPS**; LSR-YOLO \| 0.741 \| 0.649 \| 0.722 \| 0.478 \| 2114768 \| 6.6 \| 357.1; **5-Fold CV** \| 0.749 \| 0.609 \| 0.680 \| 0.493 \| 2485861 \| 7.8 \| 349.7 The precision, recall, mAP50, and mAP50-95 values show only slight variations between the original and cross-validation results, reinforcing the consistency of the LSR-YOLO model. Specifically, while there is a small increase in precision (0.741 to 0.749) and mAP50-95 (0.478 to 0.493), the recall (0.649 to 0.609) and mAP50 (0.722 to 0.680) slightly decrease, but these fluctuations are not significant enough to undermine the overall stability of the model. Furthermore, the small differences in parameters, CFLOPs, and FPS further indicate that the LSR-YOLO’s performance is robust across different data splits. |
| 8 | Missing Random Seed or Reproducibility Details: There is no mention of whether a fixed random seed was used, nor is there discussion of run-to-run variability. Reproducibility is a key part of rigor in ML research. | We have added the description of experimental reproducibility. The Settings for random seeds have been supplemented, with the default setting of 0 by the official ultralytics team, and the relevant experimental settings have also been add.  Specific modification is as follows:  (section 3.1)**--Line:375 to 378**  This study sets random seed to the default fixed integer value of 0, which controls the reproducibility of the random processes and ensures that the experimental results can be consistently replicated.  (section 3.6)**--Line:566 to 569**  The training setup is shown in Table 1. Using SGD optimizer, The model is trained with a batch size of 16, a learning rate of 0.01, and for 200 epochs. The input images are resized to 640×640 pixels by default, and momentum is set to 0.937 to accelerate convergence. |
| 9 | Grammatical Errors and Awkward Phrasing: Frequent issues with subject-verb agreement, article usage, and sentence structure.  Example:  "We uses DWConv as convolution layers..." → should be "We use DWConv as convolutional layers..."  "With only 2,114,768 parameters and 6.6 GFLOPs, LSR-YOLO is significantly more lightweight compared to advanced models including YOLOv11." → "…significantly lighter than advanced models such as YOLOv11." | We corrected the grammatical errors and inappropriate expressions throughout the entire article, enhancing its readability.  Specific modification as following:  “We use DWConv as convolutional layers...”  “…significantly lighter than advanced models such as YOLOv11.”  etc. |
| 10 | Redundancy and Wordiness: Some paragraphs are overly long and repeat information (e.g., repeating FPS comparisons in several sections).  Sentences like "This design enhances the efficiency of the model without sacrificing performance" are generic and overused. | We delete the redundant description information to make the model effect description more concise.  Specific modification is as follows:  ~~This design enhances the efficiency of the model without sacrificing performance.~~  ~~Through the analysis of experimental metrics, LSR-YOLO presents a compelling advantage in lightweight design, offering a highly efficient balance between accuracy and computational efficiency. Its reduced parameter count and GFLOPs, coupled with its high FPS, make it a robust and scalable solution for real-time object detection in various applications.~~  etc. |
| 11 | Inconsistent Terminology: Some abbreviations (e.g., “YOLOv8n-improved,” “SR-YOLO,” “LSR YOLO”) are used inconsistently or introduced without clear distinction. | Following your advice, abbreviations are used consistently.  Specific modification is as follows:  YOLOv8n-improved  LSR-YOLO |
|  | | |
| In addition to the revisions suggested by the editor and reviewers, we also corrected some errors. | | Our enhancements and refinements |
|  | YOLOv8n's training FPS on the Locount dataset was incorrectly manipulated, resulting in a result of 78.8 fps. | Due to improperly set training parameters, the previously reported YOLOv8n baseline value of 78.8 FPS was incorrect. After multiple training runs with the correct settings, we obtained the accurate baseline value of 110.4 FPS. This correction does not affect the overall results or conclusions of the paper. We have now updated the data and sincerely apologize for the oversight. Thank you. |
|  | There are grammatical errors throughout the entire text. | We have refined the sentence structures and grammar throughout the text to enhance its readability, with the revised sections highlighted for your reference. |
|  | Figures 15 and 16 are newly added figures. The results obtained through software analysis are not clear. |    (1)Original image (2) improved image  **Figure 15.** Examples of negative samples for product detection with multiple object occlusions.     (1)Original image (2) improved image  **Figure 16.** Examples of negative samples for product detection under low-light conditions. |
|  | The title of the manuscript has been optimized for improved clarity and rigor. | The optimized title is “LSR-YOLO: a Lightweight and high-Speed YOLO model for the Retail products detection” |
|  | Some details of Figure 2 in the manuscript have been changed, as shown in Figure 2 below.  The details in Figure 2 of the manuscript have been modified, as shown in the updated version below. The red-circled regions indicate the revised parts. It should be noted that the CBS module and the standard Conv operation have an inclusion relationship; CBS is not a separate type of convolution, but rather a composite structure consisting of convolution + batch normalization + activation function. In the original figure, the replacement of the CBS module by the ADown module was inaccurately illustrated. The correct depiction should indicate that ADown replaces the Conv module. The figure has been updated accordingly. This revision does not affect any of the results presented in the manuscript, as the change is solely a clarification in the diagram for greater specificity. |   **Figure 2.** Overall structure diagram of YOLOv8n-improved model for the retail product object detection |
